# Design of liquid crystalline nanoparticles: Linking composition to membrane interactions and siRNA delivery

**DOI:** 10.1016/j.ijpx.2026.100635

**Published:** 2026-08-11

**Authors:** Ana Vitória Pupo Silvestrini, Márcia Carvalho de Abreu Fantini, Ana Paula Ramos, Maria Vitória Lopes Badra Bentley

**Affiliations:** a School of Pharmaceutical Sciences of Ribeirão Preto, University of São Paulo, Ribeirão Preto, SP, Brazil; b Institute of Physics, University of São Paulo, São Paulo, SP, Brazil; c Department of Chemistry, Faculty of Philosophy, Sciences and Letters at Ribeirão Preto, University of São Paulo, Ribeirão Preto, SP, Brazil

**Keywords:** Liquid-crystalline nanoparticle, Lyotropic liquid crystal, Langmuir monolayer, Small interfering RNA, Poloxamer, Design nanoparticle

## Abstract

The clinical translation of small interfering RNA (siRNA) therapeutics critically depends on delivery systems capable of protecting nucleic acids, enabling efficient cellular uptake, and promoting cytosolic release. Liquid crystalline nanoparticles (LCNs) are promising carriers due to tunable internal nanostructure and biocompatibility, yet how composition controls membrane affinity and biological performance remains limited. Here, we compared two cationic reverse-hexagonal LCNs formulated from monoolein, oleic acid, poly(allylamine hydrochloride) and either poloxamer 407 (P407) or poloxamer 188 (P188). Both LCN-P407 and LCN-P188 shared reverse-hexagonal organization, mean diameters of 150–185 nm, low polydispersity (0.09–0.18), positive zeta potentials (10–20 mV), and protection of siRNA from RNase A. Langmuir isotherms and Brewster angle microscopy revealed pronounced adsorption and expansion of DPPC monolayers, with LCN-P188 inducing stronger perturbations, consistent with its higher cytotoxicity (∼20% increase in cell death) relative to LCN-P407. Crucially, siRNA delivered by LCN-P407 showed significantly greater uptake (> 1.7-fold) than that administered by LCN-P188. Functionally, as a proof of concept, we showed that LCN-P407-siTNFα induced robust gene silencing in LPS-stimulated macrophages, reducing TNFα secretion by 1.2–3.5-fold depending on particle concentration and incubation time. LCN-P188-siTNFα produced only delayed and modest reductions (1.3–1.6-fold). In addition, in dermatomized porcine skin, LCN-P407 also exhibited superior cutaneous penetration, delivering siRNA efficiently into the viable epidermis and dermis. Collectively, these results reveal a direct relationship between LCN composition, membrane affinity, and functional performance, providing mechanistic insight for the rational design of LCN-based carriers in RNA therapeutics.

## Highlights

• Poloxamer composition dictates LCN stability and membrane interactions.
• LCNp-P407 enhances siRNA uptake and TNFα silencing over LCNp-P188.
• LCN interfacial behavior predicts cellular toxicity and delivery efficiency.
• LCN-DPPC monolayer assays revealed stabilizer-dependent membrane perturbation.
• LCN composition modulated gene silencing kinetics in macrophages.

## Graphical abstract

**Figure:**
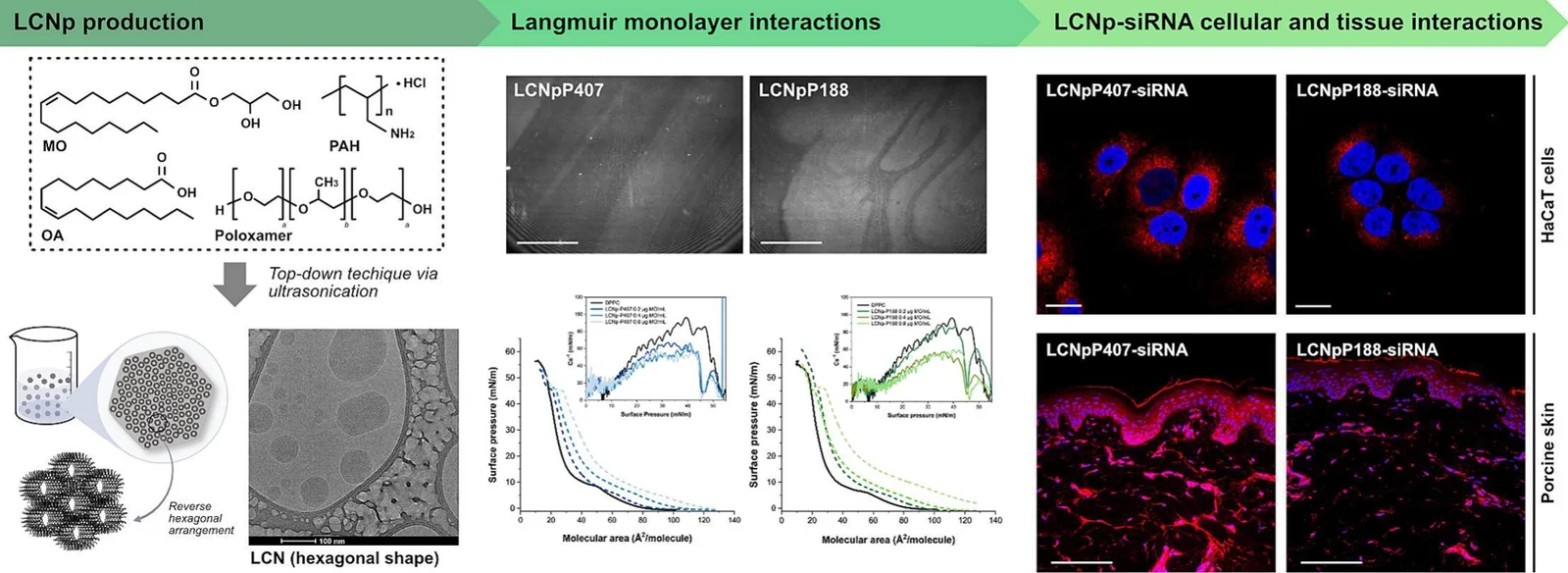

## 1 Introduction

In recent years, advances in nanomedicine and materials engineering have produced multifunctional nanoplatforms with improved potency and biocompatibility for therapeutic applications (Shan et al., 2022). Among these, non-lamellar liquid-crystalline nanoparticles (LCN), particularly reverse hexagonal and bicontinuous cubic mesophases, have attracted attention due to their highly ordered internal nanostructure, structural robustness, and ability to promote membrane fusion and cargo release (Fong et al., 2016; Mezzenga et al., 2019; Tan et al., 2019; Yu Helvig et al., 2021). The internal curvature, aqueous nanochannels, and tunable surface organization of these mesophases allow modulation of nano-bio interactions across multiple length scales (Rodrigues et al., 2019; Tan et al., 2019; Yap et al., 2024; Zhen et al., 2012).

These features are particularly relevant for nucleic acid delivery, including small interfering RNA (siRNA) (Silvestrini et al., 2024). siRNAs can transiently silence otherwise undruggable targets, and the clinical value of RNA interference has been validated by the recent approval of RNAi-based therapeutics (Corydon et al., 2023; Naeem et al., 2025). However, siRNA clinical translation remains limited by its high molecular weight and polyanionic nature, which hinder cellular uptake due to electrostatic repulsion from cell membranes, and by rapid degradation by nucleases (Dong et al., 2019; Dowdy, 2017). These barriers emphasize the need for nanoscale carriers that combine biocompatibility with controlled interfacial properties and efficient cytosolic delivery (Corydon et al., 2023; Naeem et al., 2025).

Although previous studies demonstrate that non-lamellar lipid mesophases enhance membrane fusion and facilitate endosomal escape of therapeutic RNAs, a critical knowledge gap persists (Dyett et al., 2019; Kim et al., 2018; Kim and Leal, 2015; Leal et al., 2010; Sarkar et al., 2020; Shen et al., 2011; Strachan et al., 2020; Yanez Arteta et al., 2018). The impact of steric stabilizer architecture and cationic polymer incorporation on interfacial mechanics and functional gene silencing remains poorly defined. Incorporating polycations is essential for nucleic acid loading (Mendes et al., 2022); however, the liquid-crystalline structure is highly sensitive to these molecules due to charge, packing, and responsiveness to intracellular pH, particularly in the case of cationic and ionizable lipids (Rajesh et al., 2021; Sarkar et al., 2020; Vicentini et al., 2013; Yu et al., 2024; Yu et al., 2023) Furthermore, subtle variations in poly(ethylene oxide) (PEO)/poly(propylene oxide) (PPO) block composition can alter surface corona organization, membrane insertion dynamics, and intracellular targeting, ultimately affecting biological performance..

Here, we systematically investigated how poloxamer architecture and polycation incorporation modulate the colloidal behavior, internal mesostructure, membrane insertion, and siRNA delivery efficiency of reverse hexagonal LCNs. To establish a well-defined and biologically relevant model for interfacial studies, Langmuir monolayers of 1,2-dipalmitoyl-sn-glycero-3-phosphocholine (DPPC), a major phosphatidylcholine component of mammalian cell membranes, were employed to probe nanoparticle–membrane interactions under controlled conditions. These biophysical insights were complemented by cellular uptake, functional gene silencing, and *in vitro* skin distribution analyses to provide a multiscale evaluation of delivery performance. In this context, siTNFα was used as a proof-of-concept anti-inflammatory payload to validate functional intracellular delivery. Although not restricted to a specific disease model, this strategy is particularly relevant for TNFα-driven inflammatory skin disorders (Andretto et al., 2023). Overall, our results demonstrate that stabilizer-driven interfacial organization, rather than surface charge alone, plays a central role in dictating biological performance across molecular, cellular, and tissue levels.

## 2 Methods

The list of key materials is described in the supplementary section.

### 2.1 LCN preparation

LCN were prepared by a top-down method involving hydration of a bulk mesophase followed by ultrasonication. The precursor gel was obtained at 8:1:91 (*w*/w/w) MO/OA/aqueous phase ratio, in which molten MO was mixed with OA and subsequently hydrated with 0.01 M sodium phosphate buffer (pH 7.0) containing P407 or P188 (0.5, 1 and 1.5% *w*/*v*). For cationic formulations, PAH (0.5%, w/v) was dissolved in the aqueous phase prior to hydration. After 24 h equilibration, the bulk phase was fragmented by ultrasonication (30% amplitude, 1 min, ice bath; Vibra-Cell™ VCX750 Sonics & Materials, Inc.) to yield stable dispersions.

Neutral systems were designated LCNn-P407 and LCNn-P188, while PAH-containing formulations were termed LCNp-P407 and LCNp-P188. For complexation, siRNA was incubated with cationic LCN (2:1 N/P ratio) for 30 min at room temperature.

### 2.2 LCN physical characterization

#### 2.2.1 Hydrodynamic diameter, zeta potential, and particle concentration

The intensity-weighted average hydrodynamic diameter (reported as the z-average) and the particle size distribution (reported as the polydispersity index, PdI) of LCN were analyzed by dynamic light scattering (DLS) using a Malvern Zetasizer Nano (Malvern Instruments, UK). Correlation graphs generated by the software and the correlogram intercept values (>0.9) were used to measure the reliability of the gaussian distributions obtained. Measurements of the electrophoretic mobility of the nanoparticles were expressed as zeta potential using the provided software. The means of three determinations in different batches of the same dispersion type were used in the analysis.

The LCN concentration (particle number per mL) was determined on a NanoSight NS300 (Malvern Instruments, UK). The recorded videos were analyzed using Malvern software (NTA 3.4 Build 3.4.003).

#### 2.2.2 Evaluation of siRNA binding, structural integrity, and nuclease protection in LCNp systems

The RiboGreen® assay was performed to quantify the efficiency of siRNA (20 μM) binding with LCNp, according to the manufacturer's instructions. Samples were analyzed in a spectrofluorometer (λ_exc_ 500/ λ_em_ 525 nm), and the siRNA binding efficiency was calculated by dividing the quantified free siRNA concentration by the initial siRNA concentration and multiplying the result by 100.

Complex formation (LCNp-P407 or LCNp-P188; N/P 2:1) and siRNA release were further evaluated by agarose gel electrophoresis using GelRed® under standard conditions (TAE buffer, 100 V, 110 mA, 20 min). For release and integrity analysis, complexes were treated with heparin (50 IU, 37 °C, 10 min) prior to electrophoresis, and bands were visualized under UV illumination (Transilluminator, Loccus Biotechnology, Brazil) using Quantity One software.

Protection against enzymatic degradation was investigated *via* RNase A digestion. LCNp-siRNA complexes (20 μM) were incubated with RNase A (0.5 and 5.0 μg/mL) at 37 °C for 1 and 24 h. Controls included intact siRNA, siRNA with EDTA/heparin, and RNase-treated siRNA. Enzyme activity was inactivated with EDTA (100 mM), followed by heparin-mediated siRNA release and electrophoretic analysis as described above.

Conformational stability of siRNA upon complexation was analyzed by circular dichroism using a Jasco J-810 Spectropolarimeter at 25 °C (200–350 nm, 1 mm cuvette). Spectra (average of four scans) were obtained for free siRNA (20 μM), isolated LCNp (1:50 and 1:100), heparin (50 IU), and LCNp-siRNA complexes prepared immediately prior to analysis.

#### 2.2.3 Small angle X-ray scattering (SAXS) analysis

The LCN internal mesophase was investigated by SAXS using a Xeuss® 2.0 (Xenocs, France) set-up with a Pilatus bidimensional detector, operating at a wavelength of 1.5418 Å (copper tube) and sample-to-detector distance of 0.9 m (*q* range covers 0.015 Å^−1^ and 0.43 Å^−1^). Special glass capillaries (diameter of 2.0 mm) containing the samples were placed in a temperature-controlled (23 ± 1 °C) sample holder. Measurements were performed for 0.01 M sodium phosphate buffer (pH 7.0) as background to obtain the absolute scattering intensity (Silvestrini et al., 2023). The scattering vector, *q*, was determined from the scattering angle by using the relationship *q* = (4π/λ)sinθ, with 2θ being the scattering angle, and λ being the X-ray wavelength. To identify the phase type, the *q* values of the peaks were correlated with Miller indices. Lattice parameters were calculated from peak positions, and variations greater than 0.10 nm were considered significant based on instrument precision.

#### 2.2.4 Cryogenic electron microscopy (Cryo-EM) analysis

Cryo-EM analyses were performed in a Talos F200C microscope (Thermo, USA), operating at 200 kV, with a Ceta 16 M 4 k × 4 k camera (Thermo, USA) for digital image acquisition. The lacey carbon film on a 300-mesh copper grid (Ted Pella®, USA) were previously treated with a load of 25 mA for 50 s, in an EasiGlow (I) equipment (Ted Pella®, USA) and the vitrification of samples Vitrobot Mark IV (Thermo, USA). The sample was applied to each grid, performing the excess draining step (Blot time 3 and Blot force −3) and freezing the grids immediately in liquid ethane. After this step, the grids were kept in liquid nitrogen until insertion under the microscope. ImageJ® software was used to analysis of Cryo-EM micrographs.

### 2.3 Langmuir monolayers

#### 2.3.1 Surface pressure-surface area per molecule isotherms

Langmuir experiments were performed using a KSV-Nima KN2002 trough (Biolin Scientific, Sweden) equipped with symmetric hydrophilic barriers and a Wilhelmy plate (filter paper) as the surface pressure sensor. The trough (243 cm^2^ surface area) was controlled by dedicated LB software. DPPC monolayers were formed by depositing a chloroform solution (1 mg/mL) at the air–water interface over a PBS subphase (pH 7.2) in the presence or absence of LCN. After 15 min to allow solvent evaporation, the monolayers were compressed at a constant rate of 15 mm/min at 23 ± 1 °C. Surface pressure and molecular area were measured with accuracies of 1 mN/m and 1 Å^2^, respectively. All experiments were performed at least in triplicate.

Monolayer compressibility was assessed from π–A isotherms by calculating the compressional modulus (Cs^−1^ = −A(∂π/∂A)). LCN insertion was evaluated by determining changes in molecular area (ΔArea) at defined surface pressures relative to control monolayers.

#### 2.3.2 Surface topography and morphology characterization

The morphology and texture of the DPPC monolayer on the air-water surface in real time as a function of surface pressure were studied by Brewster angle microscopy (BAM) (Roldán-Carmona et al., 2012). Experiments were performed by using KSV NIMA MicroBAM (Biolin Scientific, Sweden) coupled to the KSV-Nima trough.

### 2.4 *In vitro* cellular studies

#### 2.4.1 Cell line and culture conditions

Human immortalized non-tumorigenic keratinocytes cell line HaCaT and murine immortalized non-tumorigenic monocyte/macrophage cell line Raw264.7 (ATCC TIB-71) were maintained in DMEM supplemented with 10% FBS. Cells were grown at 37 °C and 5% CO_2_ and were split every 2–4 days and discarded after 15 passages.

#### 2.4.2 Cell viability assay

The cytocompatibility of LCN was measured by the resazurin reduction assay. Briefly, HaCaT and Raw264.7 cells (10^4^ cells/ well) were seeded in a 96-well plate and after an overnight period, the culture medium was replaced with fresh culture medium containing each LCN (concentrations: 50 to 250 μg of MO/mL). After 24 h, a solution of resazurin (25 μg/mL) in fresh culture medium was added to each well and the cells were incubated for another 4 h. Resorufin fluorescence (resazurin reduction) was measured on a BioStack Ready device (BioTek Synergy 2, USA) according to the manufacturer's conditions (λ_exc_ 530/ λ_exc_ 590 nm). Data are expressed as a percentage of viable cells compared to the untreated control.

#### 2.4.3 Cellular uptake of the LCNp-siRNA complex in 2D culture

Cellular uptake of siRNA mediated by the LCNp-siRNA complex was studied by confocal laser scanning microscopy (CLSM) and fluorescence-activated cell sorting (FACS) analysis. For FACS analysis, the cells (HaCat cell line) were seeded in a 12-well plate (5 × 10^5^ cells/ well) and after an overnight period, the culture medium was replaced with fresh culture medium containing each sample (LCNp-siRNA AF647 or naked siRNA at 80 μM). After 6, 12, or 24 h, the cells were trypsinized and centrifuged, and the resulting pellet was resuspended in PBS and subjected to BD FACSCanto™ I (BD Biosciences, US).

For CLSM image analyses, cells were seeded onto 35-mm coverglass bottom dishes (10^5^ cells/well), and samples (LCNp-siRNA AF647 or siRNA naked at 80 μM) maintained for 12 h. After that, the cells were washed with PBS, fixed with paraformaldehyde (2% *w*/*v*; 10 min) and stained with DAPI solution (0.3 μg/ mL; 10 min) for nucleus labeling. Finally, CLSM images were obtained using a Leica TCS SP8 CLSM microscope (Leica Microsystems Inc., USA) equipped with a 63× oil immersion objective and laser for DAPI (λ_exc_ 358/ λ_exc_ 461 nm) and for AlexaFluor 647 (λ_exc_ 650/ λ_exc_ 671 nm).

#### 2.4.4 Proof of concept: TNFα knockdown in LPS-stimulated cells

The gene silencing capacity of siRNA was evaluated in an acute inflammation model using siRNA targeting TNFα. For this purpose, Raw264.7 cells were seeded in a 96-well plate (2.5 × 10^4^ cells/well) and and after an overnight period, the culture medium was replaced with fresh culture medium containing LPS (1 μg/mL) and the formulations for 12, 24, and 48 h. The formulations were used at the following concentrations: naked siTNFα 150 nM; LCNp-P407 or LCNp-P188 at 1.5 or 3 × 10^8^ particles/mL with or without siTNFα 150 nM. At the end of the experiment, the supernatants were collected and TNFα levels were quantified by ELISA, following the manufacturer's instructions. All measurements were performed in quadruplicate.

### 2.5 Cutaneous distribution of the siRNA-LCN complex in porcine skin

The intensity and cutaneous distribution of LCNp-complexed siRNA AF647 (10 μM) in skin were examined by CLSM. Skin samples (dermatomized to a thickness of 500 μm), according to OECD Guideline 428 (Guidelines, 2004), were placed in a Franz Phoenix® automated vertical diffusion cell (Teledyne Hanson Research, USA). The recipient solution consisted of sodium phosphate buffer (DEPC water; pH 7.4 ± 0.2) and was maintained at 32 °C under constant agitation of 400 rpm. The naked siAF647, LCNp-P407-siAF647, and LCNp-P188-siAF647 formulations were applied to the donor compartment. After 2, 6, 12, and 24 h, the skins were collected and cryopreserved for later processing (cryostatic microtome Leica, Germany). The histological cryo-sections were stained with DAPI and CLSM images were obtained using a Leica TCS SP8 CLSM microscope (Leica Microsystems Inc., USA) equipped with a 40× oil immersion objective and laser for DAPI (λ_exc_ 358/λ_em_: 461 nm) and for AlexaFluor 647 (λ_exc_ 650/ λ_em_ 671 nm).

### 2.6 Data analysis

Data are presented as mean ± standard deviation. Statistical analyses were performed using GraphPad Prism 9. Comparisons were conducted using one-way or two-way ANOVA followed by appropriate *post hoc* tests, as indicated in figure legends. Differences were considered significant at *p* < 0.05.

## 3 Results and discussion

### 3.1 LCN characterization

In this study, LCN composed mainly of MO and OA were produced by a top-down approach using ultrasonication to disintegrate the liquid-crystalline bulk gel and form colloidal dispersions. Due to infinite expansibility, high-energy breakdown of the liquid-crystalline gel in excess aqueous phase in the presence of a stabilizer forms thermodynamically stable particles (de Campo et al., 2004). The stabilizing effect of poloxamers P407 and P188 (0.5–1.5% *w*/*v*) was first evaluated. All precursor gels exhibited typical fan-like birefringence under polarized light, with no mesophase transition after stabilizer incorporation (**Supplementary Fig. 1**).

After sonication, LCNn-P407 and LCNn-P188 formed homogeneous milky–opalescent dispersions, consistent with preserved internal nanostructure. DLS showed unimodal size distributions (**Supplementary Fig. 2**), and increasing the stabilizer concentration resulted in particles with smaller sizes, from 167 to 147 nm for LCNn-P407 and from 165 to 132 nm for LCNn-P188 (**Supplementary Table 1**). This is likely due to the reduction in the interfacial tension between lipids and water, which enhances stabilizer association with the LCN surface and promotes steric stabilization (Magana et al., 2019; Tilley et al., 2013; Zhai et al., 2015).

During long-term storage, LCNn-P407 showed superior maintenance of particle size, PdI, and zeta potential compared to LCNn-P188 (**Supplementary Fig. 3**). The superior performance of P407 likely reflects a balanced interfacial architecture: a sufficiently long PPO block to anchor at the membrane interface, combined with a hydrated PEO corona that limits excessive bilayer disruption (Chong et al., 2011). Poloxamers with longer PPO blocks and higher molecular weight exhibit greater interfacial affinity and reduced internal accommodation within the mesostructure, increasing the fraction of molecules available for surface stabilization (Chountoulesi et al., 2021; Demurtas et al., 2015; Magana et al., 2019; Tilley et al., 2013; Zhai et al., 2015). This explanation aligns with the minimal changes in lattice parameters observed in the SAXS analysis of LCNn, which will be discussed later.

Subsequently, LCNn-P407 and LCNn-P188 were further evaluated at 1% (*w*/*v*) stabilizer (12.5 wt% MO). Both systems exhibited symmetric, unimodal size distributions (PdI 0.09–0.18) and similar particle concentrations (∼3.5 × 10^12^ particles/mL) (Table 1), which agreed with previously reported values for LCN generated by the ultrasonication method (Bor et al., 2022; Rossetti et al., 2011; Silvestrini et al., 2023). The mean hydrodynamic diameter was slightly larger for LCNn-P407 (151.3 ± 0.1 nm) than for LCNn-P188 (139.6 ± 0.1 nm). This difference is consistent with stabilizer architecture, as P407 (PEO_100_/ PPO_65_) contains longer PEO chains than P188 (PEO_75_/ PPO_30_), favoring the formation of a thicker steric corona at the nanoparticle–water interface. The outward extension of these hydrated PEO chains increases the hydrodynamic radius (Demurtas et al., 2015; Shubhra et al., 2014), resulting in the slightly larger particle size observed for LCNn-P407.

**Table 1:** LCNn and LCNp colloidal properties.

Formulation	Z-average size (nm)	PdI	Zeta potential (mV)	siRNA binding (%)	Concentration (x 10^12^ nanoparticles/ mL)
*LCNn-P407*	150.3 ± 0.1	0.151 ± 0.007	−41.60 ± 0.80	n.a.	3.85 ± 0.18
*LCNp-P407*	157.8 ± 2.3	0.151 ± 0.006	+10.10 ± 1.22	n.a.	2.49 ± 0.13
*LCNp-P407-siRNA*	160.8 ± 2.5	0.182 ± 0.012	+9.64 ± 1.60	96.30 ± 0.20	n.a.
*LCNn-P188*	139.6 ± 1.1	0.121 ± 0.005	−46.12 ± 1.02	n.a.	3.55 ± 0.28
*LCNp-P188*	185.2 ± 2.1	0.090 ± 0.005	+21.60 ± 1.82	n.a.	4.07 ± 0.22
*LCNp-P188-siRNA*	187.5 ± 1.1	0.166 ± 0.013	+17.40 ± 2.23	99.65 ± 0.06	n.a.

Data are reported as means ± SD (*n* = 3/ 3 independent formulations).

Abbreviations: LCNn = liquid-crystalline nanoparticle with negative zeta potential (without PAH polymer); LCNp = liquid-crystalline nanoparticle with positive zeta potential (with PAH polymer); P407 = poloxamer 407; P188 = poloxamer 188; siRNA = small interfering RNA (20 μM); n.a. = not applicable.

Regarding surface charge, LCN typically exhibit zeta potentials between −30 and − 40 mV, mainly due to the adsorption of hydroxyl ions mediated by PEO chains and, to a lesser extent, to the ionization of terminal carboxyl groups of MO and OA at pH 7 (Rizwan et al., 2011). In our systems, increasing the stabilizer concentration led to a more negative zeta potential (−30 to −45 mV for LCNn-P407 and − 37 to −49 mV for LCNn-P188; **Supplementary Table 1**). This trend likely reflects changes in polymer surface density and chain conformation. According to the Gouy–Chapman model, lower surface density allows greater chain mobility and a transition from a brush-like to a mushroom-like configuration, shifting the slipping plane and altering the measured zeta potential (Shubhra et al., 2014).

Since LCN inherently exhibit a negative surface charge, PAH, a biodegradable cationic polymer rich in protonable amine groups (NH_2/_ NH_4_^+^) at physiological pH, was incorporated to enable electrostatic complexation with siRNA and facilitate intracellular delivery (Andreozzi et al., 2017; Corydon et al., 2023; Di Silvio et al., 2019; Han et al., 2015; Silvestrini et al., 2024). Based on previous optimization, 0.5% (*w*/*v*) PAH provided homogeneous dispersions and conferred a positive zeta potential suitable for siRNA binding (Silvestrini et al., 2023). Importantly, PAH incorporation preserved the reverse hexagonal mesophase in the liquid-crystalline bulk (**Supplementary Fig. 1, photomicroscopy iv and viii**). While LCNp-P407 maintained colloidal parameters comparable to neutral systems, LCNp-P188 displayed a larger hydrodynamic diameter (185.2 ± 2.1 nm) and lower PdI (0.090 ± 0.005). Consistent with previous reports, incorporating cationic polymers or lipids often increases LCN particle size, depending on the concentration used (Deshpande and Singh, 2017; Rajesh et al., 2021; Yu et al., 2024). Importantly, LCNp-P188 dispersions showed a high positive zeta potential (approximately +20 mV), likely reflecting enhanced PAH mobility within the diffuse layer due to the shorter PEO chains of P188.

**Fig. 1:**
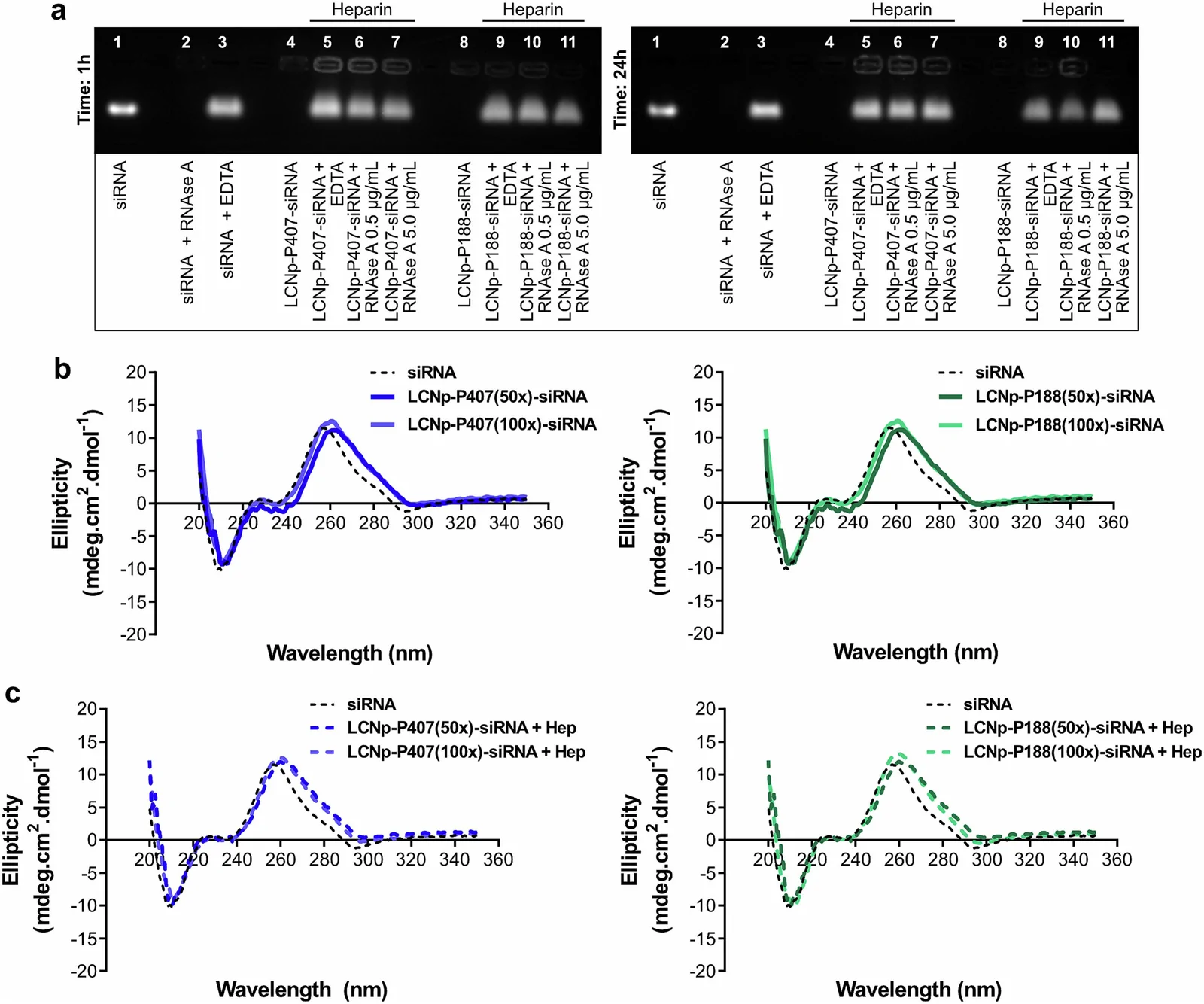
Characterization of LCNp-siRNA complexes. **(a)** Electrophoretic mobility of naked siRNA (naked, complexed with LCNp-P407 and LCNp-P188; released from complexes after competition with heparin and after protection assay with RNase A). **(b)** Overlay of the CD spectra of naked siRNA and complexed with LCNp-P407 or LCNp-P188 (diluted 50 or 100×). **(c)** Overlay of the CD spectra of naked siRNA, and siRNA released from LCNp-P407 or LCNp-P188 after competition with heparin.

Next, incubation with 20 μM siRNA, both LCNp formulations exhibited a slight decrease in zeta potential (1–2 mV) and a modest increase in hydrodynamic diameter (2–3 nm), while preserving low polydispersity (PdI < 0.19), indicating that complexation did not compromise colloidal stability. RiboGreen® assay demonstrated >96% complexation efficiency. Electrophoretic mobility assays confirmed strong electrostatic association. As shown in Fig. 1a (lane 4 of each gel), the absence of free siRNA bands up to 24 h indicated efficient charge neutralization and inhibition of anodal migration. However, upon incubation with heparin (a polyanion), the siRNA was released from the LCNp-siRNA complex, as evidenced by the reappearance of the siRNA band. The band intensity and migration distance of naked siRNA in solution (containing heparin and EDTA – Fig. 1 **a**, lane 3) and after release of the LCNp-siRNA complexes were similar (Fig. 1 **a**, lane 5 of each gel), indicating that LCNp readily released the siRNA without altering its double-stranded structure at all times evaluated.

Given that nuclease-mediated degradation remains a major limitation in RNAi therapeutics (Silvestrini et al., 2024), the protective capacity of LCNp was assessed using RNAse A digestion followed by heparin-induced release. In Fig. 1a (lanes 6 and 7 of each gel), the released siRNA exhibited similar intensity and migration patterns to untreated siRNA (lane 3), whereas naked siRNA incubated with RNAse A was completely degraded (lane 2). These results highlight the robust protective effect of LCNp on siRNA over 24 h. Although siRNA released from LCNp-P188 showed slightly reduced intensity compared to LCNp-P407, this remained stable across all time points. Given that siRNA has a diameter of 2–3 nm, it can occupy the aqueous channels within the hexagonal mesostructure, interacting with PAH in these regions (Kim et al., 2018; Svintradze and Mrevlishvili, 2005). Furthermore, it is likely that the long branches of PAH and stabilizers (P407 and P188) protect the siRNA on the outer surface against nuclease activity by steric impediment (Uz et al., 2015).

The supramolecular interactions between siRNA and LCNp were further investigated by circular dichroism (CD) spectroscopy. As shown in Fig. 1b, naked siRNA displayed the characteristic A-form helical signature, with a positive band at 264 nm associated with base stacking interactions and a negative band centered at 210 nm corresponding to the A-helix backbone conformation (Reina et al., 2018). Upon complexation with LCNp-P407 or LCNp-P188, the overall spectral profile was preserved, indicating maintenance of the A-form geometry. However, a slight decrease in molar ellipticity and a modest bathochromic shift were observed (Fig. 1**, c**), suggesting subtle adjustments in the local chiral environment of the nucleobases. These changes likely reflect electrostatic interactions between siRNA and PAH within the LCNp matrix, leading to minor conformational rearrangements without significant disruption of base pairing or stacking interactions. Such modest structural modulation could influence parameters such as duplex rigidity or hydration state, yet does not appear to compromise structural integrity.

Importantly, siRNA released after heparin competition exhibited CD spectra overlapping with those of naked siRNA (Fig. 1c), confirming recovery of the native A-helix conformation. This structural preservation was consistent with electrophoretic mobility results obtained under identical dilution and complexation conditions (**Supplementary Fig. 4**), supporting the conclusion that duplex base pairing remained largely intact during binding and release. Together, these results suggest that complexation within LCNp induces reversible and non-disruptive supramolecular interactions.

After we determined the colloidal parameters and confirmed the LCNp-P407 or LCNp-P188 ability to form a complex with siRNA, we stored LCNp-P407 and LCNp-P188 for 90 days at room temperature and monitored their parameters (**Supplementary Table 2**). The mean hydrodynamic diameter of LCNp-P407 increased gradually by approximately 30 nm from the 30th to the 90th day, which was accompanied by an increase in PdI (from 0.15 to 0.23). On the other hand, the LCNp-P188 mean hydrodynamic diameter increased after the 60th day, while PdI increased progressively after storage for 15 days. Still, values below 0.3 indicate a narrow, homogeneous particle size distribution of the LCN. Fluctuations in zeta potential values were observed. LCNp-P407 maintained a positive surface charge above +10 mV, while LCNp-P188 averaged +16 mV.

### 3.2 Characterization of the internal structure and shape of LCN

**Fig. 2:**
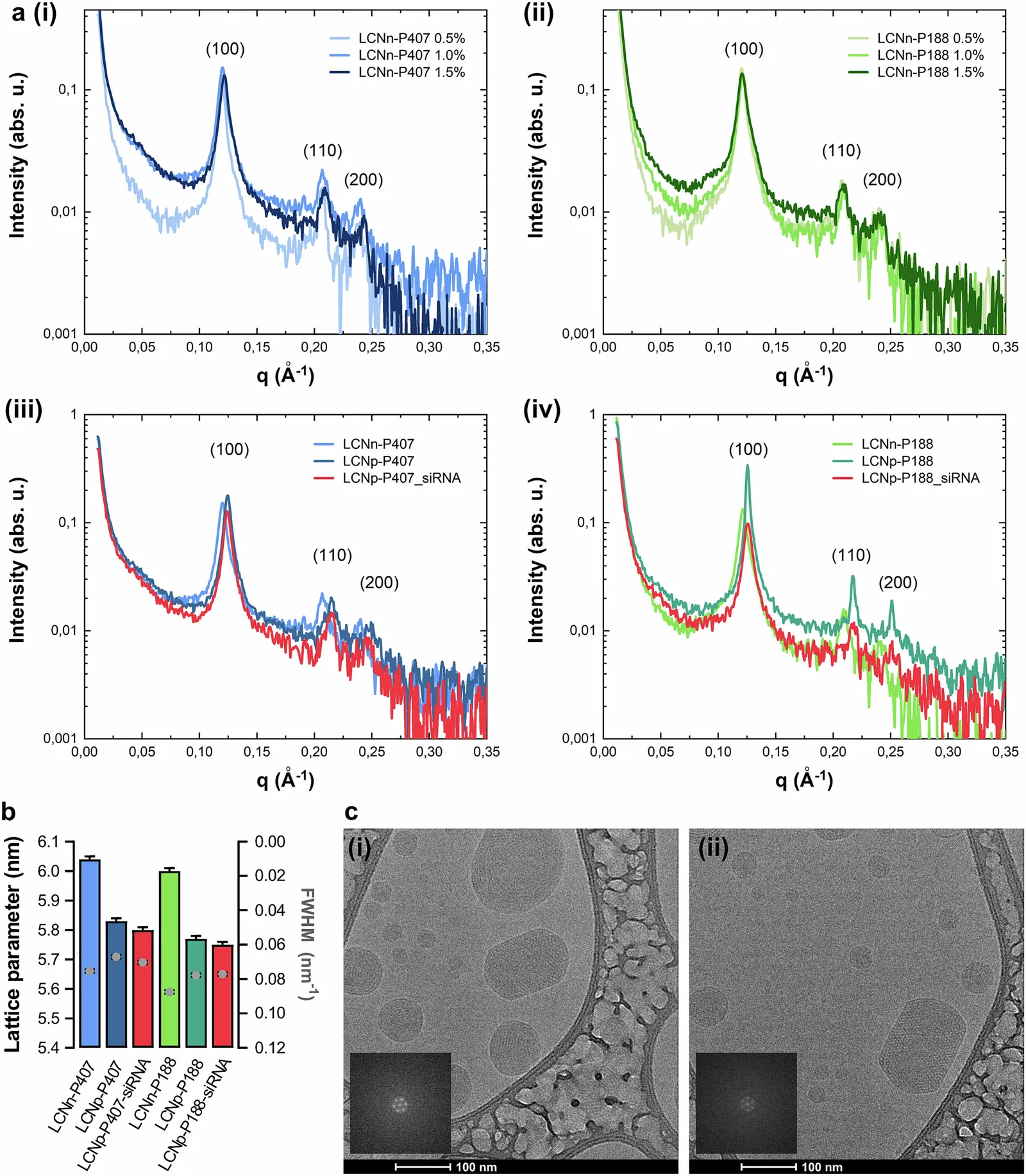
**(a)** SAXS data analysis: diffraction patterns of (i) LCNn-P407, (ii) LCNn-P188, (iii) LCNp-P407/LCNp-P407-siRNA, and (iv) LCNp-P188/LCNp-P188-siRNA together with the Bragg peaks indexed to reverse hexagonal mesophase. **(b)** Lattice parameter (bars) and FWHM (symbols) values of LCNn, LCNp, and LCNp-siRNA complexes. **(c)** Cryo-EM micrograph of LCN: (i) LCNp-P407, and (ii) LCNp-P188. The inset shows the FFT of the internal area of the particles and is used for the determination of the liquid crystalline structure, independently confirmed by SAXS analysis. Scale bar: 100 nm.

SAXS experiments were performed to analyze the internal structure of LCN and confirm that the reverse hexagonal mesophase was preserved after the bulk gel ultrasonication process. Fig. 2a shows the 1D scattering patterns and integrated intensities as a function of *q* for each LCN. All LCN formulations displayed three Bragg reflections at relative positions 1, √3, and √4, indexed to the (100), (110), and (200) planes, respectively, confirming a 2D reverse hexagonal symmetry. The formation of this mesophase at room temperature was promoted by OA, whose C18 acyl chain matches that of MO, facilitating efficient packing and promoting a wedge-shaped molecular geometry that favors negative interfacial curvature (Fong et al., 2012; van ‘t Hag et al., 2017).

Lattice parameters, calculated from reciprocal spacing, and FWHM values were used to assess structural organization. Increasing concentrations of P407 or P188 induced only subtle reductions in lattice parameters (≤0.04 nm) (Fig. 2 **a, i and ii; Supplementary Table 3**), suggesting limited dehydration of MO headgroups, likely due to competition with water molecules. Consistent with previous reports, P407 appears to remain predominantly in the aqueous phase with minimal insertion into the MO/OA interface, thereby preserving the reverse hexagonal structure (Amar-Yuli et al., 2007; Nakano et al., 2002). Both stabilizers therefore mainly adsorb at the nanoparticle surface, providing steric stabilization without disrupting mesophase symmetry. However, higher stabilizer concentrations increased FWHM values, particularly for LCNn-P188 (0.07–0.09), indicating a moderate rise in liquid-crystalline disorder. (**Supplementary Table 3**).

LCNp-P407 and LCNp-P188 exhibited reductions in lattice parameters (0.21–0.23 nm), as evidenced by Bragg peak shifts toward higher *q* values (Fig. 2 **a, iii and iv;** and Fig. 2 **b**), indicating contraction of the hexagonal unit cell after PAH incorporation. This effect is attributed to partial dehydration of the internal aqueous channels, which increases hydrocarbon chain mobility, enhances negative interfacial curvature, and reduces lattice spacing (Amar-Yuli et al., 2007; Mishraki-Berkowitz et al., 2017). In contrast to other cationic polymers or aminolipids, such as polyetherimide, oleylamine, or quaternary ammonium lipids, which can induce mesophase transitions (*e.g.*, cubic-to-hexagonal or hexagonal-to-micellar) due to increased headgroup size and electrostatic repulsion (Rajesh et al., 2021; Sarkar et al., 2020; Vicentini et al., 2013), PAH did not significantly alter the reverse hexagonal symmetry. This suggests that its relatively hydrophilic nature and lower density of ionized primary amines limit perturbation of monolein spontaneous curvature, thereby preserving lipid packing (Kulkarni et al., 2016).

As expected, upon siRNA complexation in LCNp, the characteristic of the hexagonal phase was maintained, with only marginal additional reductions in lattice parameter. Such subtle changes in unit cell dimensions are consistent with nucleic acid incorporation in cationic lipid systems (Kim and Leal, 2015; Leal et al., 2010). Furthermore, because siRNA molecules are a very short double-stranded rod (typically 20 base pairs) and 2–3 nm in diameter, they accommodate in liquid crystalline domains without causing effects on lipid curvature (Kim et al., 2018; Silvestrini et al., 2023), supporting its localization in the water channels, but in close contact with the reverse micelles in their 2D hexagonal organization, presumably due to the electrostatic attraction by the PAH amines.

In addition to this data, cryo-EM was performed to obtain more information about the shape of LCNp-P407 and LCNp-P188. Fig. 2c shows nanoparticles in the range of 80 to 200 nm with the shape of elongated hexagons and some quasi-spherical particles, which is in agreement with previous reports (Magana et al., 2019; Urandur et al., 2020). No morphological differences were observed between P407- and P188-stabilized systems. Internal hexagonal ordering, confirmed by FFT patterns and the presence of curved striations, further supported reverse hexagonal symmetry (Akhlaghi et al., 2016; Fong et al., 2010; Mat Azmi et al., 2015; Yu Helvig et al., 2021). These striations are commonly attributed to the deformation of hexagonally arranged water cylinders. This configuration can be adopted to better stabilize the LCN, allowing for a uniform hydrophobic surface. Occasionally, in some LCN that show hexagonally compacted water, cylinders are sometimes observed on the surface, demonstrating that the latter configuration is also possible (Gustafsson et al., 1997; Sagalowicz et al., 2006; Yaghmur et al., 2005).

### 3.3 Interaction between LCN and Langmuir monolayers

#### 3.3.1 Influence of the stabilizer molecular structure on surface pressure-surface area isotherms

DPPC was selected as a zwitterionic model membrane to provide a controlled and well-defined reference system for comparing the interfacial effects of LCN composition. By employing a single-component phospholipid with a neutral net charge at physiological pH, this approach minimizes electrostatic contributions and avoids the additional complexity introduced by anionic lipids (Moehwald and Brezesinski, 2016; van Meer et al., 2008). Consequently, it enables a more direct and mechanistic assessment of stabilizer-dependent membrane perturbation, isolating the role of the nanocarrier surface properties.

**Fig. 3:**
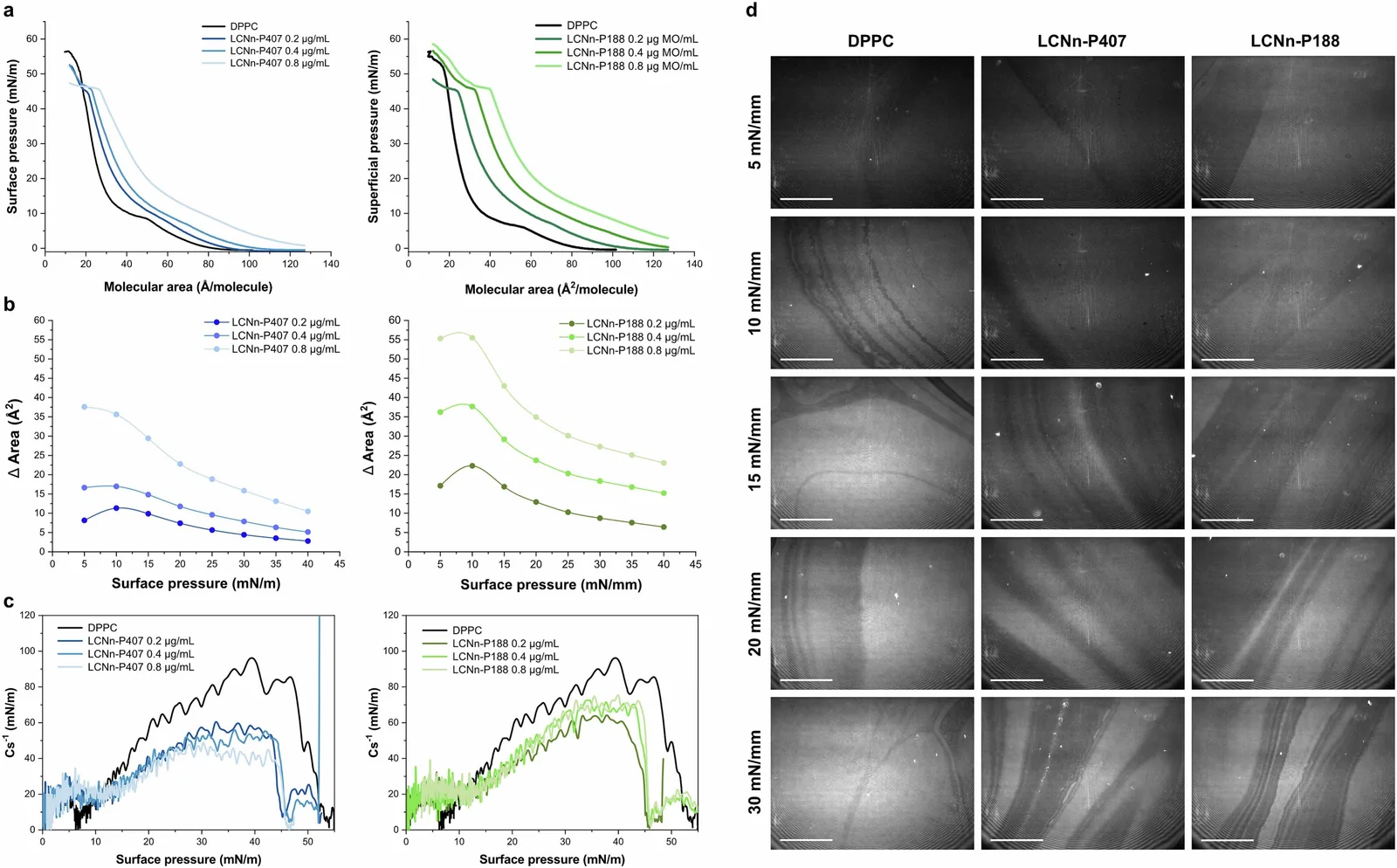
Negatively charged LCN interact with DPPC monolayers. **(a)** Surface pressure *versus* surface area isotherms; **(b)** Δ area calculated from the area occupied per lipid on the surface minus the area occupied per lipid in the subphase containing LCNn at different concentrations; **(c)** Compressional modulus (Cs^−1^) *versus* surface pressure for DPPC monolayers in the PBS subphase (black line); LCNn-P407 at 0.2 μg (dark blue line), 0.4 μg (blue line), and 0.8 μg of MO/mL (light blue line), and LCNn-P188 at 0.2 μg (dark green line), 0.4 μg (green line), and 0.8 μg of MO/mL (light green line). **(d)** BAM images of DPPC as a function of the surface pressure in the different subphases: PBS, LCNn-P407 (0.2 μg of MO/mL) and LCNn-P188 (0.2 μg of MO/mL). Light regions correspond to thicker (condensed) domains. Scale bar: 100 μm. (For interpretation of the references to colour in this figure legend, the reader is referred to the web version of this article.)

Within this simplified and controlled framework, we then investigated how the negatively charged LCNn-P407 and LCNn-P188 interact with lipid interfaces representative of biological membranes. Specifically, their interactions with DPPC monolayers were evaluated through π–A isotherms obtained in a Langmuir trough. Different concentrations of LCNn-P407 or LCNn-P188 were introduced into the aqueous subphase beneath the DPPC monolayer. LCNn-P407 and LCNn-P188 presented surface activity, *i.e.*, π remained unaltered at the concentrations used in the absence of the DPPC monolayers. However, they interacted with DPPC, to modify the π-A isotherm profile (Fig. 3**, a**): LCNn-P407 and LCNn-P188 expanded the DPPC monolayers, indicating that they penetrated among the lipids. Addition of increasing LCNn-P407 or LCNn-P188 concentration to the subphase enhanced the DPPC monolayer.

Changes in the minimum molecular area occupied per lipid (Δ Area) in the presence or absence of LCN as a function of monolayer compacting (π) can be useful to discuss the area occupied by the LCN at the air-liquid interface **(**Fig. 3**, b**). LCNn-P407 and LCNn-P188 presented positive Δ Area, indicating that the LCN were present at the interface. Moreover, they were not expelled from the DPPC monolayers even at high π, indicating their high affinity for DPPC. The larger Δ area calculated for LCNn-P407 and LCNn-P188 could be correlated with the ability of LCNn-P188 to interact with the monolayer due to the smaller hydrophilic portion present in the P188 structure and greater exposure of the liquid-crystalline structure of the LCN. Thus, it is suggested that the outer layer provided by P407 (with a greater number of PPO blocks) may limit the affinity with the monolayer. LCN insertion into the DPPC monolayers reduced Cs^−1^ compared to pure DPPC (Fig. 3**, c**), thus increasing monolayer compressibility and changing lipid organization to a more fluid state (Vollhardt and Fainerman, 2006).

The adsorption of the LCN into the DPPC monolayers was also followed by images from a BAM (Fig. 3**, d**). The presence of bright spots with increased π was related to formation of condensed DPPC lipid domains (Daear et al., 2017). In the presece of LCNn-P407 or LCNn-P188, we visualized dark fields crossing the bright fields, especially at π higher than 15 mN/m. This confirmed the LCNn-P407 or LCNn-P188 affinity for the DPPC monolayers and the presence of LCNn at the air-liquid interface even at high π.

#### 3.3.2 Influence of LCN positive surface charge and siRNA loaded-LCN on surface pressure-surface area isotherms

**Fig. 4:**
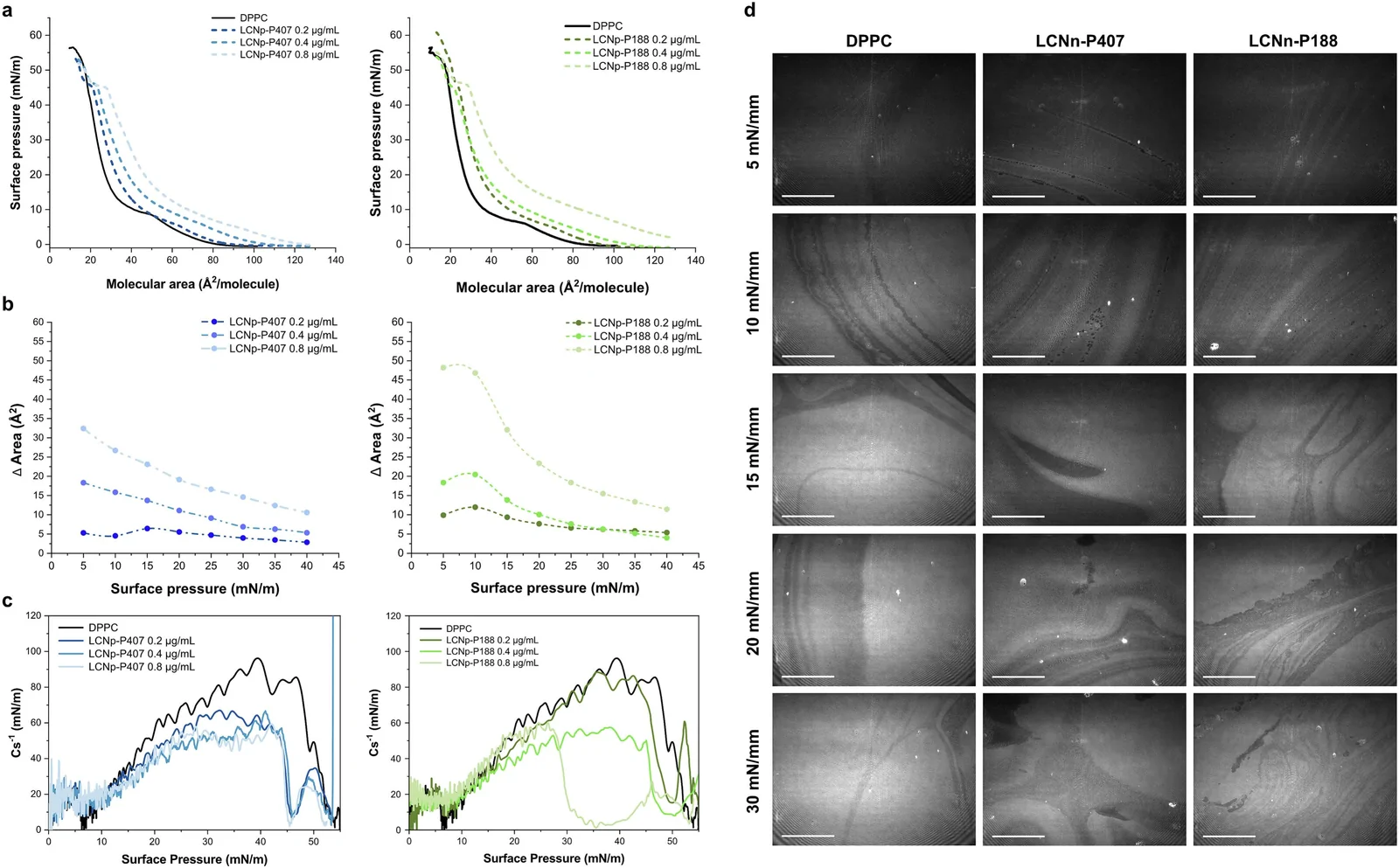
Positively charged LCN interact with DPPC monolayers. **(a)** Surface pressure *versus* surface area isotherms; **(b)** Δ area calculated from the area occupied per lipid on the surface minus the area occupied per lipid in the subphase containing LCNp at different concentrations; **(c)** Compressional modulus (Cs^−1^) *versus* surface pressure for DPPC monolayers in the subphase composed of PBS (black line); LCNp-P407 at 0.2 μg of MO/mL (dark blue line), 0.4 μg of MO/mL (blue line), and 0.8 μg of MO/mL (light blue line), and LCNp-P188 at 0.2 μg of MO/mL (dark green line), 0.4 μg of MO/mL (green line), and 0.8 μg of MO/mL (light green line); **(d)** BAM images of DPPC as a function of the surface pressure in the different subphases: PBS, LCNp-P407 (0.2 μg of MO/mL) and LCNp-P188 (0.2 μg of MO/mL). Light regions correspond to thicker (condensed) domains. Scale bar: 100 μm. (For interpretation of the references to colour in this figure legend, the reader is referred to the web version of this article.)

The π-A isotherms obtained for DPPC in the presence of LCNp-P407 and LCNp-P188 behaved similarly (*i.e.*, monolayer expansion to larger molecular areas) to their negatively charged counterparts (Fig. 4**, a**). This could be attributed to the DPPC zwitterionic polar head, which can interact with positively or negatively charged groups. The positive Δ Area values (Fig. 4**, b**) confirmed that the LCN remained inserted in the monolayers at higher pressure, which supported their high affinity for the DPPC molecules. As in the case of LCNn-P407 and LCNn-P188, LCNp-P188 presented higher Δ Area than LCNp-P407, indicating larger extent of adsorption at the air-liquid interface. Reduced Cs^−1^ revealed that the monolayer organization changed to a more fluid state in the presence of the LCN (Fig. 4**, c**).

Regarding the LCN applications and interaction with lipids, the data obtained from the Langmuir monolayers confirmed that the systems could be used as versatile deliverers of drugs and other compounds to cells through interaction and changes in biomembrane fluidity.

The adsorption of the positively charged LCN (LCNp-P407 and LCNp-P188) onto the DPPC monolayers was also followed by BAM images (Fig. 4**, d**). As in the case of LCNn-P407 and LCNn-P188, there were dark fields crossing the bright fields, especially at π higher than 15 mN/m. This confirmed the affinity of the LCN for the DPPC monolayers and the presence of LCNp-P407 and LCNp-P188 at the air-liquid interface even at high π.

**Fig. 5:**
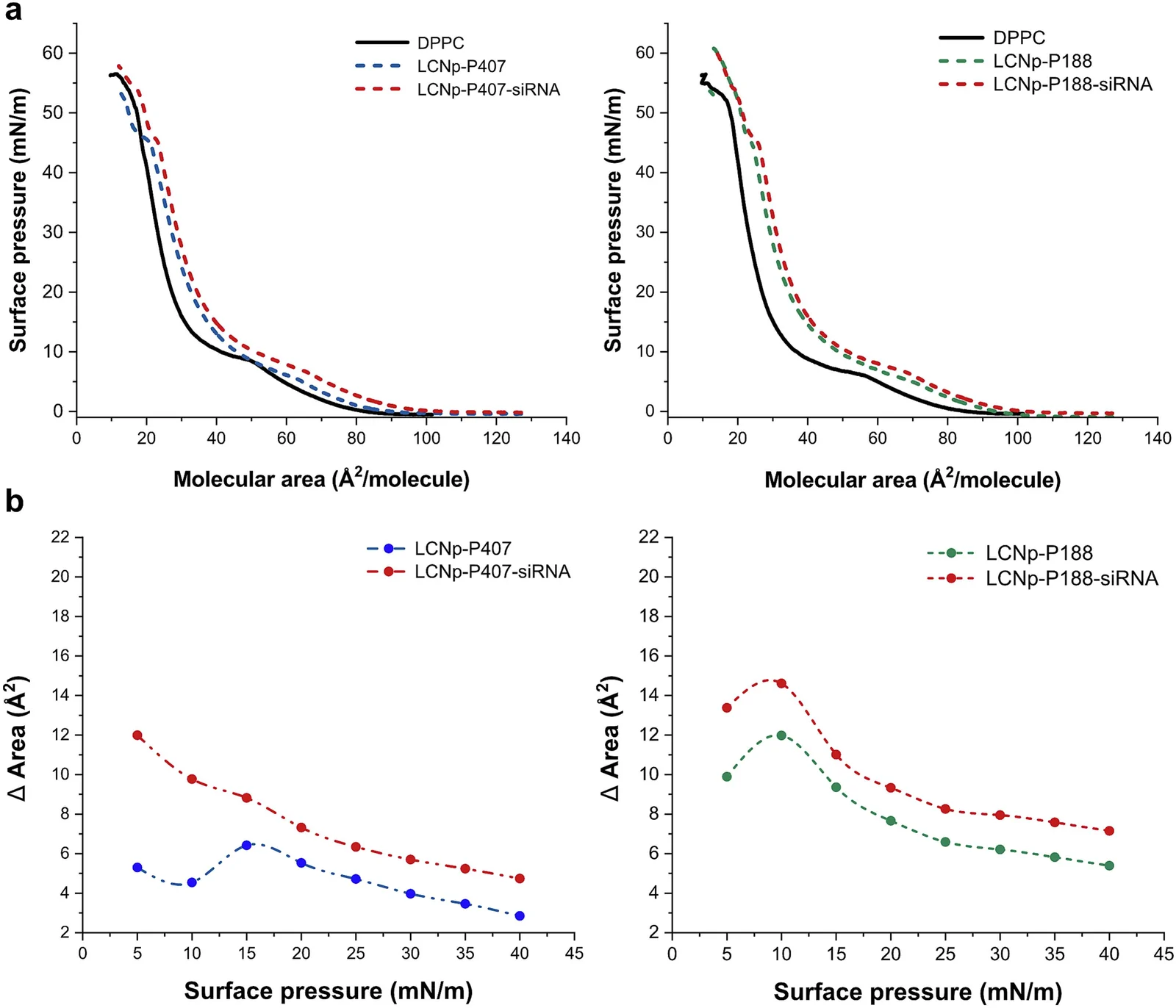
LCNp containing siRNA interact with DPPC monolayers. **(a)** Surface pressure *versus* area isotherms; **(b)** Δ area calculated from the area occupied per lipid on the surface minus the area occupied per lipid in the subphase containing LCNp or LCNp-siRNA in the subphase composed of PBS (black line); LCNp-P407 (blue dashed line), LCNp-P407-siRNA (red dashed line), LCNp-P188 (green dashed line), and LCNp-P188-siRNA (orange dashed line). The LCNp concentration was 0.2 μg of MO/mL, and the siRNA concentration was 10 μM. (For interpretation of the references to colour in this figure legend, the reader is referred to the web version of this article.)

We verified how siRNA addition to LCNp-P407 or LCNp-P188 affected their interaction with DPPC by means of π-A isotherms (Fig. 5). For this, the lower LCNp-P407 or LCNp-P188 concentration (0.2 μg/mL) was used for comparison. The isotherm profiles remained unaltered after siRNA was incorporated into the LCNp-P407 or LCNp-P188 structure **(**Fig. 5, red and blue lines and red and green lines). Moreover, the slightly higher Δ Area values obtained in the presence of siRNA could support higher adsorption of LCNp-P407 or LCNp-P188 into the DPPC monolayers.

### 3.4 LCN-siRNA complex cellular and tissue interactions

The findings above demonstrate that stabilizer architecture (PEO/PPO block length) and surface charge modulate LCN colloidal behavior and interfacial interactions. Both anionic and cationic LCN interacted with DPPC monolayers, a model membrane system, supporting the biological relevance of these interfacial effects. At the cellular level, such physicochemical attributes are expected to influence cytocompatibility, membrane interaction, internalization, and intracellular delivery efficiency. These aspects are particularly critical for siRNA, which is highly susceptible to endonuclease degradation and exhibits limited membrane permeability, thus requiring structurally optimized carriers for therapeutic performance (Dilliard and Siegwart, 2023; Silvestrini et al., 2024). Therefore, elucidating how LCN composition governs cell–material interactions is essential for rational vector design.

**Fig. 6:**
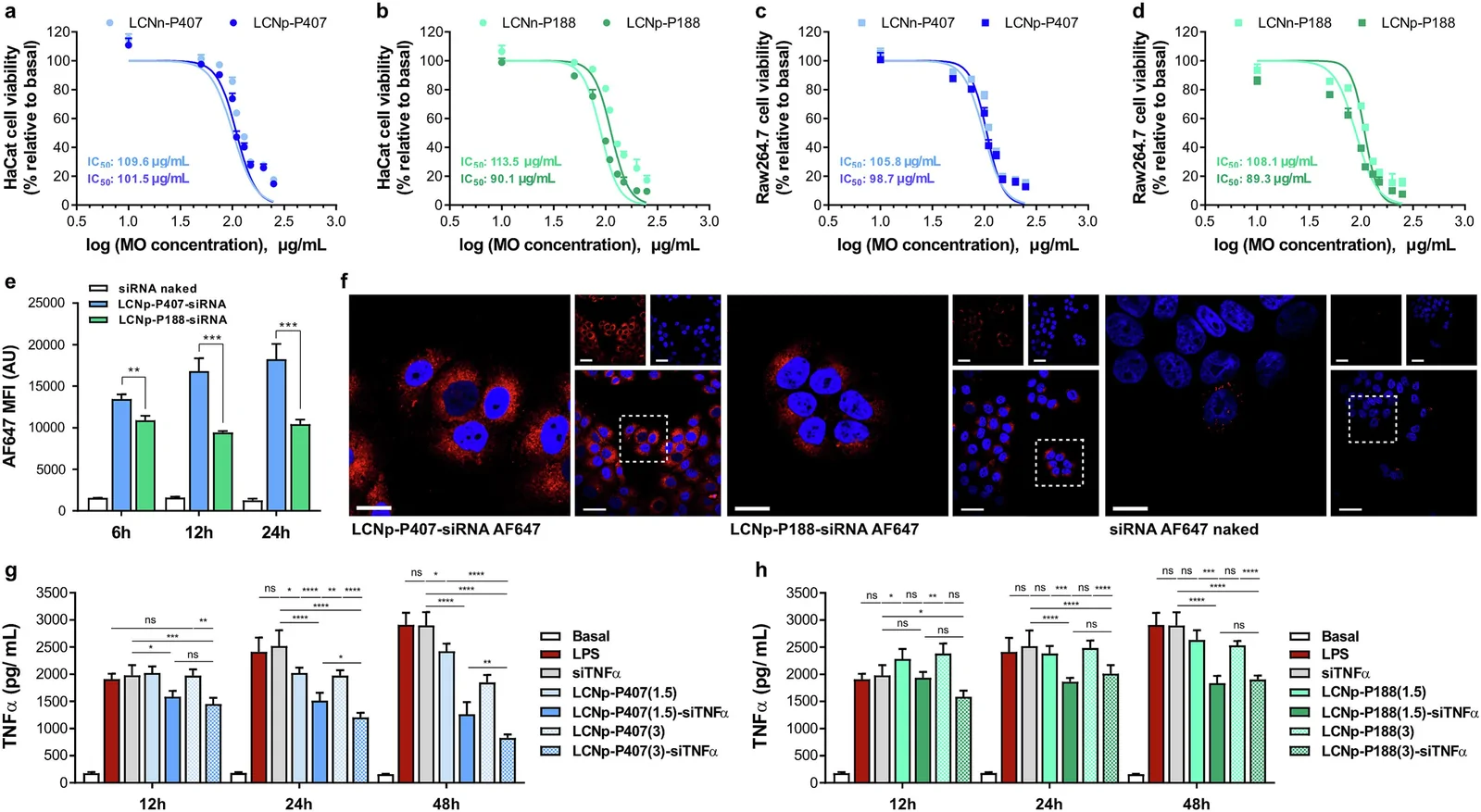
Cellular internalization of the siRNA-LCNp complex and TNFα knockdown *in vitro*. **(a-d)** Logarithmic dose-viability curve of IC_50_ after 24 h of treatment. Data shown are means ± SD (*n* = 8). **(e)** siRNA AF647 mean fluorescence intensity measured by FACS in HaCat cells after treatment with siRNA AF647 naked or complexed in LCNp. Data shown are means ± SD (*n* = 3/ 3 independent tests); Student's *t*-test (** *p* = 0.001 and *** *p* = 0.0003). **(f)** CLSM photomicroscopies of HaCat cells treated with siRNA AF647 naked or complexed in LCNp for 12 h. Nuclei represented in blue (DAPI) and siRNA in red (AF647). Scale bar: 50 μm (zoom: 10 μm). **(g-h)** Levels of TNFα secreted by Raw264.7 cells stimulated with LPS and treated simultaneously with the formulations. DMEM culture medium and LPS (500 ng/ mL) were used as negative control and positive control, respectively. Data shown are means ± SD (*n* = 4). Two way-ANOVA, Tukey's post test: * *p* < 0.05; ** *p* ≤ 0.01; *** *p* ≤ 0.001 and **** *p* < 0.0001. (For interpretation of the references to colour in this figure legend, the reader is referred to the web version of this article.)

Prior to uptake studies, cytotoxicity was evaluated in HaCaT and Raw264.7 cells over a range of concentrations (μg MO/mL). Dose-response curves (Fig. 6a-d) showed a concentration-dependent decrease in viability, with IC_50_ values between 90 and 110 μg MO/mL for all formulations. To comply with ISO 10993-5 criteria (>70% viability), subsequent assays were conducted at concentrations below 50 μg MO/mL. Cationic formulations (LCNp-P407 and LCNp-P188) exhibited greater cytotoxicity than their anionic counterparts, with LCNp-P188 being the most potent (100 μg MO/mL: 52% viability for LCNp-P188 *vs.* 74% for LCNp-P407). This enhanced cytotoxicity likely reflects stronger electrostatic and hydrophobic interactions between positively charged surfaces and cellular membranes, consistent with the DPPC monolayer findings. Such interactions may facilitate insertion into the lipid bilayer core, promoting membrane destabilization, an effect previously associated with increased toxicity in liquid crystalline nanoparticles (Shen et al., 2011, Shen et al., 2010). In agreement with literature reports, formulations with low or near-neutral surface charge tend to display improved tolerability, regardless of nanostructure or cationic lipid composition (Chou et al., 2014; Zhen et al., 2012).

Beyond superficial charge, surface architecture and stabilizer distribution (external steric PEO corona *vs.* PPO interfacial anchoring) control interfacial adsorption and lipid mobility, thereby influencing cell-particle interactions, uptake routes and downstream metabolic effects (Deshpande and Singh, 2017; Dong et al., 2011; Tilley et al., 2013). The interplay between polymer surface coverage and its penetration into the liquid-crystalline matrix modulates membrane perturbation and cytotoxicity (Tan et al., 2019), while formulation parameters (fatty-acid composition, mesophase structure) and cell lineage further influence biological responses (Rodrigues et al., 2019; Tran et al., 2018, Tran et al., 2015). Enhanced steric shielding (longer PEG/PEO chains) generally reduces membrane contact and toxicity by limiting polymer insertion, whereas insufficient coverage may promote bilayer disruption (Zhai et al., 2015). Although the molecular mechanisms regulating stabilizer organization remain incompletely defined, their elucidation is critical for the rational design of safer nanocarriers.

Next, cellular uptake was assessed using AF647-labeled siRNA under sub-cytotoxic conditions (8 μg MO/mL; ∼3 × 10^8^ nanoparticles/mL; >90% viability). As shown in Fig. 6e, both formulations enhanced siRNA uptake in HaCaT cells; however, LCNp-P407 consistently promoted 1.2- to 1.7-fold higher internalization than LCNp-P188, whose mean fluorescence intensity exhibited minor fluctuations over time. CLSM images (Fig. 6**, f**) corroborate these findings, showing intense red fluorescence distributed throughout the cytoplasm and perinuclear region, particularly prominent in cells receiving LCNp-P407-siRNA. Importantly, cell morphology remained largely preserved, indicating that both formulations were well tolerated at the tested concentration.

The superior performance of LCNp-P407 suggests that cellular internalization is governed not solely by surface charge but by integrated interfacial properties, including corona organization, hydrophobic balance, and nanoparticle mechanics (Deshpande and Singh, 2017; Murgia et al., 2010; Tran et al., 2015; Yap et al., 2025). Subtle variations in surface architecture can influence endocytic routing and intracellular trafficking (Deshpande and Singh, 2017). While P188-stabilized systems may favor stronger hydrophobic membrane adsorption, potentially limiting productive cytosolic release, the longer PPO segment of P407 may promote more favorable membrane interactions and trafficking profiles associated with enhanced delivery efficiency (Silvestrini et al., 2023; Tan et al., 2019; Yang et al., 2021). These observations align with previous reports showing that moderately cationic systems can outperform strongly cationic formulations in functional siRNA delivery (Zhen et al., 2012). Moreover, poloxamers with extended PPO segments, such as P407, can actively modulate membrane dynamics and trafficking processes, enhancing internalization and gene silencing efficiency (Alakhova and Kabanov, 2014; Yang et al., 2021).

To further assess the functional impact of siRNA delivery by LCNp, we employed TNFα knockdown in LPS-stimulated Raw 264.7 macrophages as a proof-of-concept model. TNFα is an important mediator of the inflammatory immune response, and controlling inflammation is useful for treating both autoimmune and autoinflammatory syndromes (Andretto et al., 2023). LPS triggers robust secretion of TNFα, a key pro-inflammatory cytokine involved in multiple innate immune pathways. As shown in Fig. 6g, at all evaluated time points, LCNp-P407-siTNFα produced a 1.2- to 3.5-fold reduction in TNFα secretion, at both 1.5 × 10^8^ and 3 × 10^8^ nanoparticles/mL. Notably, the LCNp-P407 carrier alone also exhibited a modest immunomodulatory effect at 24 h, consistent with the presence of oleic acid, a fatty acid with recognized antioxidant and anti-inflammatory properties (Lin et al., 2019). Increasing the particle density of LCNp-P407 to 3 × 10^8^ nanoparticles/mL yielded an even greater TNFα suppression (1.2- to 1.5-fold additional reduction) over time, suggesting an additional contribution.

In contrast, TNFα suppression in cells treated with LCNp-P188-siTNFα emerged only at later time points (24 and 48 h), with reductions of 1.3–1.6-fold and no significant differences between the two particle concentrations tested (Fig. 6**, h**). This delayed and less pronounced response is consistent with the lower intracellular siRNA accumulation previously observed for LCNp-P188. Overall, LCNp-P407 demonstrated markedly superior functional delivery, achieving >40% reduction in TNFα secretion at 24 h compared with LCNp-P188. These findings reinforce the notion that the interfacial properties conferred by P407, particularly its PPO-mediated membrane insertion and favorable endocytic routing, translate into more efficient cytosolic siRNA release and, consequently, more potent gene silencing.

**Fig. 7:**
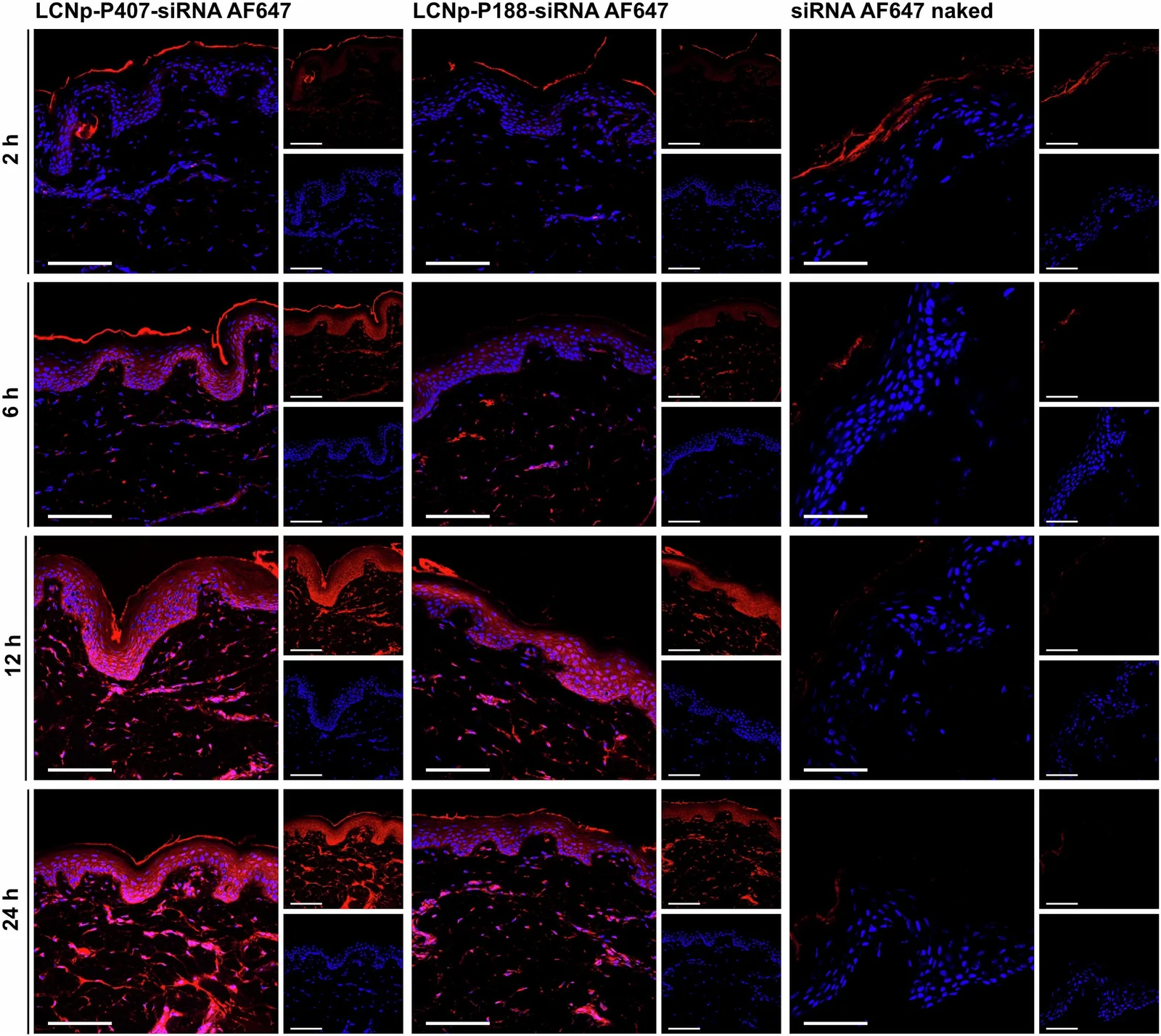
Representative CLSM images of cutaneous distribution of siRNA using dermatomized porcine skin. siRNA AF647 (red – Alexa Fluor 647) and nuclei (blue - DAPI). Scale bar corresponds to 100 μm. (For interpretation of the references to colour in this figure legend, the reader is referred to the web version of this article.)

Finally, to explore extrahepatic, non-invasive siRNA delivery, dermatomized porcine skin was employed as a physiologically relevant *in vitro* model to assess topical distribution. To this end, Franz diffusion cell assays were performed to simulate cutaneous administration and evaluate the ability of the formulations to penetrate beyond the skin surface and reach viable layers. The CLSM images in Fig. 7 show that both LCNs increased siRNA fluorescence in the stratum corneum, and epidermis compared to naked siRNA. Over time, signal intensity increased in the viable epidermis and dermis, reaching a maximum at 24 h. LCNp-P407-siRNA consistently produced higher fluorescence than LCNp-P188-siRNA. The enhanced transdermal delivery likely reflects a combination of factors: nanometric size (greater contact area), MO-mediated penetration enhancement (hydrolysis to oleic acid and glycerol increasing hydration and fluidity, leading to lower diffusional resistance), and stabilizer-dependent lipid interactions (Leekumjorn et al., 2009; Lopes et al., 2007; Rowat et al., 2006). Specifically, a higher density of PPO blocks in P407 may promote lipid perturbation and stratum corneum fluidization, facilitating siRNA passage into viable layers (Morris et al., 2022).

## 4 Conclusion

This study demonstrates how nonionic block copolymers of PEO and PPO from the poloxamer class influence the colloidal properties and interactions of LCN and LCN-siRNA complexes with biomembranes across molecular, cellular, and tissue scales. We successfully produced reverse hexagonal mesophase LCN with sizes below 200 nm and narrow PdI. Among them, LCN-P407 exhibited greater colloidal stability over time compared to LCN-P188. The incorporation of the cationic polymer, PAH, preserved the colloidal characteristics and hexagonal mesostructure while providing an efficient positive surface charge (10–20 mV), enabling electrostatic interactions with siRNA phosphate groups.

In Langmuir monolayers of DPPC, used as a model of biological membranes, both LCN-P407 and LCN-P188 interacted with phospholipids. Besides PPO length, the longer PEO chain of P407 likely increases the hydrated corona thickness, modifies the slipping plane, and contributes to the distinct membrane-interaction profile observed for the two poloxamers. LCN-P188 induced greater fluidization and alterations, correlating with the observed cytotoxic profile in 2D cell monolayers *in vitro*. In contrast, LCNp-P407 exhibited superior cytocompatibility, accompanied by significantly higher cellular uptake (1.2–1.7-fold) and enhanced cytoplasmic/perinuclear siRNA distribution without compromising cell morphology. Functionally, LCNp-P407-siTNFα induced a robust and dose-responsive reduction in TNFα secretion (1.2–3.5-fold), whereas LCNp-P188-siTNFα produced delayed and modest effects. In *ex vivo* porcine skin, both systems penetrated beyond the stratum corneum, but LCNp-P407 yielded markedly higher siRNA-associated fluorescence in deeper layers. For topical siRNA, delivery beyond the stratum corneum into viable epidermis is often sufficient for epidermal targets, whereas deeper dermal deposition may be advantageous for dermal targets.

Collectively, these findings underscore that stabilizer-driven interfacial organization, not surface charge alone, governs biological performance. Integrating membrane interaction studies with mechanistic cellular evaluation is therefore essential for the rational design of LCN platforms optimized for efficient and safe gene delivery. In addition, taken together, the data identify P407 as the preferred stabilizer for this platform, because it combines adequate membrane interaction with lower cytotoxicity, greater colloidal stability, and superior functional delivery.

## CRediT authorship contribution statement

**Ana Vitória Pupo Silvestrini:** Writing – original draft, Validation, Software, Methodology, Investigation, Formal analysis, Conceptualization. **Márcia Carvalho de Abreu Fantini:** Writing – review & editing, Software, Methodology. **Ana Paula Ramos:** Writing – review & editing, Software, Resources. **Maria Vitória Lopes Badra Bentley:** Writing – review & editing, Supervision, Project administration, Funding acquisition, Conceptualization.

## Ethics declaration

Not applicable.

## Funding

This work was developed within the framework of National Institute of Science and Technology of Pharmaceutical Nanotechnology (INCT-Nanofarma), and supported by 10.13039/501100001807São Paulo Research Foundation (FAPESP, Brazil, grant #2014/50928-2 and #2025/26972-6) and “10.13039/501100003593Conselho Nacional de Desenvolvimento Científico e Tecnológico” (CNPq, Brazil, grant #465687/2014-8 and #408769/2024-6). A.V.P.S. was FAPESP fellowship (grant #2022/01969–4).

## Declaration of competing interest

The authors declare the following financial interests/personal relationships which may be considered as potential competing interests:

Maria Vitoria Lopes Badra Bentley reports equipment, drugs, or supplies was provided by State of Sao Paulo Research Foundation. Maria Vitoria Lopes Badra Bentley reports equipment, drugs, or supplies was provided by National Council for Scientific and Technological Development. If there are other authors, they declare that they have no known competing financial interests or personal relationships that could have appeared to influence the work reported in this paper.

## Data Availability

All the data reported in this work are available upon request.
