# Supplementary material for: Design of liquid crystalline nanoparticles: Linking composition to membrane interactions and siRNA delivery
Source: Int J Pharm X. 2026 Aug 11;12:100635. doi: 10.1016/j.ijpx.2026.100635 (PMC13499094; doi:10.1016/j.ijpx.2026.100635)
Supplement: Supplementary file 1 [file mmc1.docx]

**Design of liquid crystalline nanoparticles: linking composition to membrane interactions and siRNA delivery**

Ana Vitória Pupo Silvestrini^1^; Márcia Carvalho de Abreu Fantini^2^; Ana Paula Ramos^3^; Maria Vitória Lopes Badra Bentley^1^*

**SUPPLEMENTARY MATERIALS AND METHODS:**

***1 Materials***

Monoolein (MO; Myverol^®^ 18-99k; consists of 82% monoacylglycerols, 86.6% of which is monoolein) was kindly supplied by Kerry Group (Ireland). Oleic acid (OA), poloxamer 407 (P407), poloxamer 188 (P188), poly(allylamine) hydrochloride (PAH; 17.5 kDa molecular weight), 4′, 6-diamidine-2′-phenylindole dihydrochloride (DAPI) dye, RNAse A solution, Dulbecco's Modified Eagle Medium (DMEM), fetal bovine serum (FBS), resazurin and lipopolysaccharide (LPS) from *Salmonella enterica* sorotype *typhimurium* were obtained from Sigma-Aldrich (USA). 1.2-dipalmitoyl-sn-glycero-3-phosphocholine (DPPC; >99% purity) was purchased from Avanti Polar Lipids (USA). Tissue-Tek_®_ O.C.T. was purchased from Sakura Finetek (USA). Sodium heparin (5000 IU/mL) was purchased from Cristália (Brazil). The GelRed^®^ dye was purchased from Biotium, Inc. (USA). RNAse-free water, *Silencer*^®^ negative control siRNA and *Silencer^®^* pre-engineered siTNFα (sense sequence CGUCGUAGCAAACCACCAATT and antisense sequence UUGGUGGUUUGCUACGACGTG) were obtained from Ambion^TM^ (USA). The AlexaFluor^TM^ 647-labeled AllStars Negative Control siRNA (siAF647) was purchased from Qiagen^®^ (GER). Quant-iT™ RiboGreen^®^ RNA Reagent and mouse ELISA kit were obtained from Invitrogen^TM^ (Thermo Fisher Scientific Inc.). All the aqueous solutions were prepared by using ultrapure water with a resistivity of 18.2 MΩ·cm from a Millipore Simplicity water purification system (pH = 7.4). All the analytical reagents were directly used without further purification.

***2 Polarized light microscopy***

The bulk liquid-crystalline mesophases were analyzed using an optical microscope with circular polarized light (Axioplan 2 Image Pol microscope, Carl Zeiss, Oberkochen, Germany) equipped with an LD-AchroPlan 32x/0.40 objective set, fitted with a video camera Axiocan HRc (Carl Zeiss AG, Germany). The systems were observed at room temperature.

**SUPPLEMENTARY RESULTS:**

**TABLES:**

**Table 1. Colloidal properties of LCNs**

| ***Formulation*** | ***Z-average size (nm)*** | ***PdI*** | ***Zeta potential (mV)*** |
| --- | --- | --- | --- |
| *LCNn-P407 0.5%* | 167.1 ± 0.9 | 0.148 ± 0.004 | -30.8 ± 0.7 |
| *LCNn-P407 1.0%* | 151.3 ± 0.6 | 0.151 ± 0.005 | -41.6 ± 0.6 |
| *LCNn-P407 1.5%* | 147.8 ± 1.7 | 0.143 ± 0.005 | -45.6 ± 0.3 |
| *LCNn-P188 0.5%* | 165.9 ± 0.6 | 0.189 ± 0.005 | -38.0 ± 1.3 |
| *LCNn-P188 1.0%* | 139.6 ± 0.9 | 0.101 ± 0.003 | -43.7 ± 0.6 |
| *LCNn-P188 1.5%* | 132.3 ± 1.1 | 0.145 ± 0.004 | -48.8 ± 0.9 |

Data are represented by mean ± standard deviation (n=3/3 independent tests).

Abbreviations: LCNn = liquid-crystalline nanoparticle with negative zeta potential (without PAH polymer); P407 = poloxamer 407; P188 = poloxamer 188.

**Table 2.** **Physical stability profile of LCN with PAH polymer over storage at room temperature for 90 days**

| **Time (days)** | **Z-average size (nm)** | | **PdI** | | **Zeta potential (mV)** | |
| --- | --- | --- | --- | --- | --- | --- |
|  | *LCNp-P407* | *LCNp-P188* | *LCNp-P407* | *LCNp-P188* | *LCNp-P407* | *LCNp-P188* |
| *1* | 158.8 ± 1.7 | 203.2 ± 1.6 | 0.151 ± 0.005 | 0.090 ± 0.004 | 10.10 ± 0.91 | 21.60 ± 0.62 |
| *15* | 162.0 ± 2.8 | 212.3 ± 2.6 | 0.164 ± 0.005 | 0.113 ± 0.005 *** | 10.60 ± 0.46 | 18.00 ± 0.24 * |
| *30* | 165.6 ± 1.6 * | 198.6 ± 1.3 | 0.155 ± 0.003 * | 0.126 ± 0.002 **** | 12.32 ± 0.27 | 18.90 ± 1.01 |
| *45* | 169.9 ± 3.0 ** | 206.1 ± 2.2 | 0.170 ± 0.006 **** | 0.214 ± 0.004 **** | 10.80 ± 0.68 | 17.42 ± 0.90 ** |
| *60* | 197.9 ± 1.8 *** | 220.2 ± 2.4 ** | 0.210 ± 0.003 **** | 0.226 ± 0.001 **** | 12.64 ± 0.78 | 18.60 ± 0.87 |
| *90* | 199.9 ± 2.4 *** | 225.6 ± 1.2 *** | 0.228 ± 0.002 **** | 0.220 ± 0.001**** | 9.70 ± 0.44 | 16.40 ± 0.84 ** |

Data shown are means ± SD (n = 3/ 3 independent formulations). Statistical analysis was determined by One-way ANOVA, Dunnett post hoc test: **** *p <* 0.0001; *** *p <* 0.0002; ** *p <* 0.004, and * *p <* 0.01.

**Table 3. Data obtained by SAXS analysis for NLCn with different concentrations of P407 and P188**

| **Formulation** | **Ratio between peaks** | **Lattice parameter (nm) ± 0.01** | **FWHM (nm^-1^)** |
| --- | --- | --- | --- |
| *LCNn-P407 0.5%* | 1: √3: √4 | 6.08 | 0.0645 |
| *LCNn-P407 1%* | 1: √3: √4 | 6.04 | 0.0753 |
| *LCNn-P407 1.5%* | 1: √3: √4 | 5.96 | 0.0769 |
| *LCNn-P188 0.5%* | 1: √3: √4 | 6.05 | 0.0729 |
| *LCNn-P188 1%* | 1: √3: √4 | 6.00 | 0.0875 |
| *LCNn-P188 1.5%* | 1: √3: √4 | 6.00 | 0.0903 |

Abbreviations: LCNn = liquid-crystalline nanoparticle with negative zeta potential (without PAH polymer); P407 = poloxamer 407; P188 = poloxamer 188; FWHM = full width at half maximum.

**FIGURES:**

**Fig. 1. Polarized light microscopy micrographs of the bulk inverse hexagonal mesophase:** (i, ii, iii) MO:AO:aqueous phase (8:1:90, w/w/w) with P407 0.5, 1.0 and 1.5%, respectively; (iv) MO:AO: aqueous phase (8:1:90, w/w/w) with P407 1.0% and PAH 0.5%; (v, vi, vii) MO:AO:aqueous phase (8:1:90, w/w/w) with P188 0.5, 1.0 and 1.5%, respectively; and (viii) MO:AO:aqueous phase (8:1:90, w/w/w) with P188 1.0% and PAH 0.5%. Temperature at 25 ºC and objective 32×.

**Fig 2.** Representative dynamic light scattering (DLS) intensity distribution of LCNn containing different concentrations of P407 or P188 (0.5, 1 and 1.5 w/v).

**Fig. 3.** Colloidal stability of LCNn with different concentrations of P407 and P188. Z-average size, PdI and zeta potential measurements of **(a,b)** LCNn-P407 and **(c,d)** LCNn-P188. Data are represented by mean ± standard deviation (n=3/3 independent formulations). Statistical analysis was determined by One-way ANOVA, Dunnett post hoc test: **** *p <* 0.0001; *** *p <* 0.0002; ** *p <* 0.004, and * *p <* 0.01.

**Fig. 4.** Electrophoretic mobility of (1) naked siRNA, (2–3) siRNA complexed with LCNp-P407 50x or 100x, (4–5) siRNA complexed with LCNp-P188 50x or 100x, (6) siRNA plus heparin, and siRNA released from (7–8) LCNp-P407 50x or 100x and (9–10) LCNp-P188 50 or 100x. siRNA at 10 µM in all the experiments.
